# CYLD Regulates RhoA Activity by Modulating LARG Ubiquitination

**DOI:** 10.1371/journal.pone.0055833

**Published:** 2013-02-06

**Authors:** Yunfan Yang, Lei Sun, Tala, Jinmin Gao, Dengwen Li, Jun Zhou, Min Liu

**Affiliations:** 1 Tianjin Key Laboratory of Protein Science and Department of Genetics and Cell Biology, College of Life Sciences, Nankai University, Tianjin, China; 2 Tianjin Key Laboratory of Medical Epigenetics and Department of Biochemistry, Basic Medical College, Tianjin Medical University, Tianjin, China; Beatson Institute for Cancer Research Glasgow, United Kingdom

## Abstract

Rho family guanosine triphosphatases (GTPases), such as RhoA, Cdc42, and Rac1, play a fundamental role in various cellular processes. The activation of Rho proteins is catalyzed by guanine nucleotide-exchange factors (GEFs), which promote the exchange of GDP for GTP. The precise mechanisms regulating the activation of Rho proteins are not fully understood. Herein, we demonstrate that RhoA activity is regulated by cylindromatosis (CYLD), a deubiquitinase harboring multiple functions. In addition, we find that RhoA-mediated cytoskeletal rearrangement, chromosome separation, and cell polarization are altered in CYLD-depleted cells. Mechanistically, CYLD does not interact with RhoA; instead, it interacts with and deubiquitinates leukemia-associated RhoGEF (LARG). Our data further show that CYLD-mediated deubiquitination of LARG enhances its ability to stimulate the GDP/GTP exchange on RhoA. These data thus identify LARG as a new substrate of CYLD and provide novel insights into the regulation of RhoA activation. Our results also suggest that the LARG-RhoA signaling pathway may play a role in diverse CYLD-mediated cellular events.

## Introduction

Rho family proteins represent a major group of small guanosine triphosphatases (GTPases) of the Ras superfamily. To date, over 20 Rho GTPases have been identified, and the most extensively studied are RhoA, Cdc42, and Rac1 [1]. As with many other small GTPases, Rho proteins cycle between a GDP-bound inactive state and a GTP-bound active state, thereby enabling signal transduction from a wide variety of membrane receptors into cells [1], [2]. As intracellular molecular switches, Rho proteins play a critical role in many cellular processes, including cell proliferation, polarization, adhesion, and motility [1], [2]. The activation of Rho proteins is mediated by guanine nucleotide-exchange factors (GEFs), which stimulate the exchange of GDP for GTP [3]. Aberrant activation of Rho proteins can have severe consequences and has been implicated in the pathogenesis of human diseases such as cancer and mental retardation [4], [5].

RhoA is the first identified Rho family GTPase, and activated RhoA regulates many aspects of cell behavior primarily by triggering downstream signaling pathways and remodeling of cytoskeletal components [2]. Leukemia-associated RhoGEF (LARG), originally isolated from a patient with acute myeloid leukemia [6], is one of the key GEFs mediating RhoA activation and belongs to the regulator of G protein signaling (RGS) domain-containing GEFs, together with p115RhoGEF and PDZ-RhoGEF [7]. However, the molecular machinery governing RhoA activation, including the action of LARG in the GDP/GTP exchange on RhoA, remains to be fully elucidated. In this study, we provide the first evidence that LARG-mediated activation of RhoA is modulated by cylindromatosis (CYLD), a deubiquitinase involved in the regulation of nuclear factor κB (NF-κB) and several other signaling pathways [8]–[10]. The CYLD-LARG axis thus constitutes a novel molecular mechanism regulating RhoA activation.

## Materials and Methods

### Materials

Antibodies against α-tubulin, CYLD and ubiquitin (Abcam), RhoA and Rac1 (Cell Signaling Technology), β-actin, γ-tubulin, Flag, Myc, and His (Sigma-Aldrich), LARG, p115RhoGEF, and PDZ-RhoGEF (Santa Cruz Biotechnology), and GFP (Roche) were purchased from the indicated sources. Horseradish peroxidase-conjugated anti-mouse and anti-rabbit secondary antibodies were obtained from Amersham Biosciences. Fluorescein- or rhodamine-conjugated secondary antibodies were from Jackson ImmunoResearch Laboratories. Nocodazole, cytochalasin D, and 4′,6-diamidino-2-phenylindole (DAPI) were from Sigma-Aldrich, and mant-GDP and mant-GTP were from Cytoskeleton.

### Plasmids

Plasmids expressing CYLD, CYLD shRNA, GFP-CYLD, GFP-CYLD-C/S, Flag-LARG, and Myc-LARG were described previously [11]–[14]. Plasmids expressing Flag-RhoA and Flag-Traf2 were constructed by insertion of their cDNAs into the pCMV-Tag2B vector. The bacterial expression plasmid for GST-RhoA was constructed by insertion of RhoA cDNA into the pGEX6P3 vector. GST-RhoA was expressed in the BL21 (DE3) strain of Escherichia coli and purified using glutathione-Sepharose 4B beads according to the manufacturer's instruction (Promega).

### Cell culture and transfection

293T cells were cultured in Dulbecco's modified Eagle's medium supplemented with 10% fetal bovine serum at 37°C in a humidified atmosphere with 5% CO_2_. Plasmids were transfected to cells by using the polyethyleneimine reagent (Sigma-Aldrich).

### Immunoblotting and immunoprecipitation

Protein samples were resolved by SDS-PAGE and transferred onto polyvinylidene difluoride membranes (Millipore). Immunoblotting was then performed as described previously [15]. For immunoprecipitation, cell lysates were incubated with antibody-conjugated agarose beads at 4°C for 2 hours. The beads were washed extensively and boiled in the SDS loading buffer, and the proteins were examined by SDS-PAGE and immunoblotting.

### Examination of active Rho proteins

The GST-RBD (Rho binding domain of Rhotekin fused to glutathione-S-transferase) and GST-PBD (p21-binding domain of Pak1 fused to glutathione-S-transferase) pulldown assays were used to detect active GTP-bound forms of RhoA and Rac1, respectively. In brief, cell lysates were incubated with GST-RBD or GST-PBD coupled glutathione sepharose beads. The beads were washed extensively, and proteins bound on beads were examined by immunoblotting with anti-RhoA and anti-Rac1 antibodies.

### Fluorescence microscopy

Cells grown on glass coverslips were fixed with 4% paraformaldehyde for 30 minutes at room temperature followed by permeabilization in 0.5% Triton X-100/phosphate-buffered saline (PBS) for 20 minutes and blocked with 2% bovine serum albumin in PBS. Cells were incubated with primary antibodies, and then with fluorescein- or rhodamine-conjugated secondary antibodies followed by staining with DAPI for 5 minutes. Coverslips were mounted with 90% glycerol in PBS and examined with an Axio Observer A1 fluorescence microscope (Carl Zeiss Inc).

### Scratch wound assay

Confluent monolayers of cells cultured in 24-well plates in the serum-free medium were scratched with a 20 µL pipette tip to create the wound. The detached cells were removed by washing with PBS, and the complete culture medium was then added to allow cell polarization/migration towards the wound. The percentage of polarized cells at the wound margin was then quantified as described previously [16].

### Cytoskeleton depolymerization and regrowth

Microtubule depolymerization and regrowth were performed by a nocodazole-washout approach as described previously [12]. To examine microfilament depolymerization and regrowth, cells were treated with 1 µg/mL cytochalasin D for 1 hour to depolymerize microfilaments, and the drug was then washed out and microfilaments were allowed to regrow.

### RhoGEF exchange assay

For analysis of GDP release from RhoA, purified GST-RhoA was loaded with fluorescent mant-GDP at 25°C for 1 hour and then incubated with Flag-LARG immunoprecipitates from 293T cells. The change in GDP fluorescence was measured with a SpectraMax fluorescence reader (Molecular Devices), and the percentage of remaining GDP-bound RhoA was calculated. To measure the GDP/GTP exchange, GST-RhoA preloaded with GDP was incubated with Flag-LARG immunoprecipitates in a GEF reaction buffer containing 0.75 µM mant-GTP, and the percentage of GTP-bound RhoA was determined by measuring the change in GTP fluorescence.

## Results and Discussion

**Figure 1:**
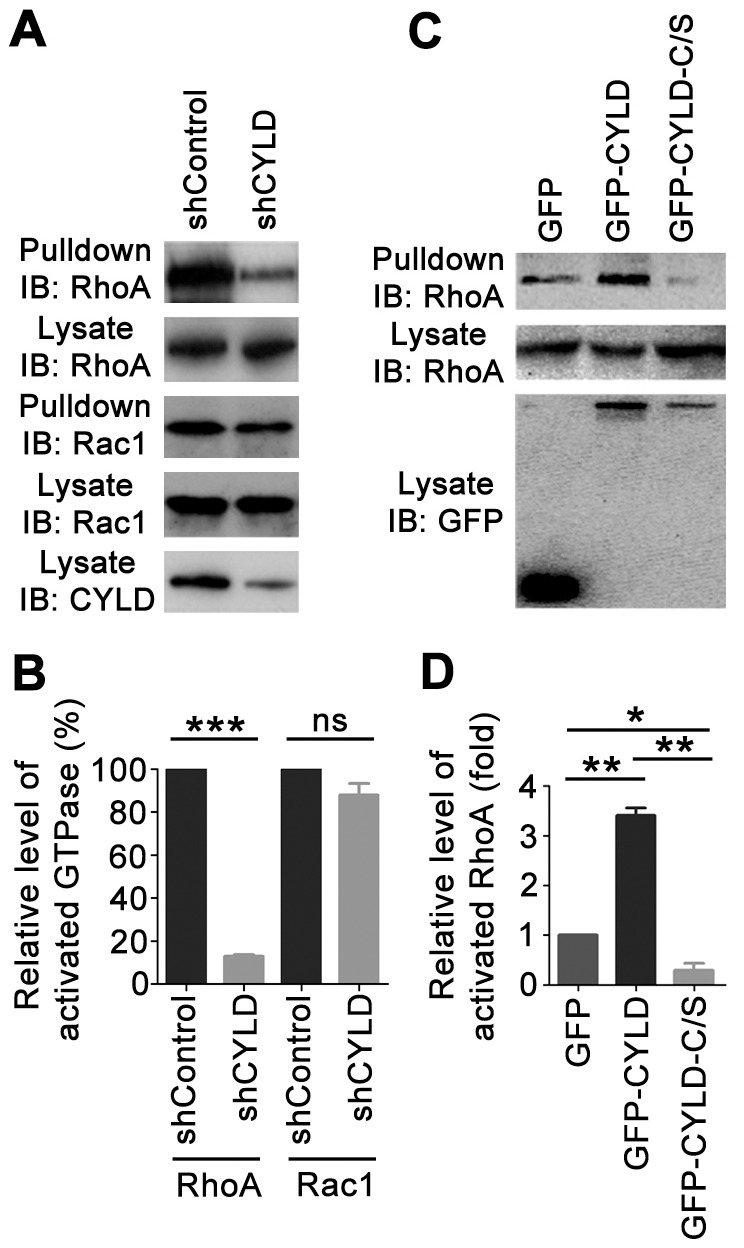
CYLD promotes RhoA activity in a deubiquitinase activity-dependent manner. (A) 293T cells were transfected with control or CYLD shRNA, and the levels of active GTP-bound RhoA and Rac1 were examined by immunoblot analysis of the GST-RBD and GST-PBD pulldown preparations. (B) Experiments were performed as in panel A, and the level of activated GTPase was determined as the ratio of GTP-bound GTPase to total GTPase. (C) Cells were transfected with GFP, GFP-CYLD, or GFP-CYLD-C/S, and the level of GTP-bound RhoA was examined as in panel A. (D) Experiments were performed as in panel C, and the level of activated RhoA was determined. *, *p*<0.05; **, *p*<0.01; ***, *p*<0.001; ns, not significant (*p*≥0.05).

Overexpression of CYLD has previously been shown to cause an accumulation of multinucleated cells [11], [17], a phenomenon also found in cells expressing constitutively active Rho family GTPases [18], [19]. In addition, CYLD has been demonstrated to regulate endothelial cell migration and angiogenesis partly by triggering Rac1 [13]. These findings prompted us to investigate whether CYLD modulates other Rho family members. We transfected 293T cells with a small hairpin RNA (shRNA) against CYLD and then examined the activated forms of Rho proteins. As shown in Fig. 1A and 1B, shRNA-mediated knockdown of CYLD expression resulted in a modest decrease in the level of activated Rac1, consistent with the previous findings [13]. CYLD shRNA dramatically inhibited the level of activated RhoA (Fig. 1A and 1B), but did not obviously affect the activation of Cdc42 (data not shown).

To assess whether the deubiquitinase activity of CYLD is involved in RhoA activation, we examined the level of activated RhoA in cells transfected with GFP, GFP-CYLD, or GFP-CYLD-C/S (a catalytically inactive C601S mutant). In agreement with the CYLD shRNA result, overexpression of GFP-CYLD promoted RhoA activity (Fig. 1C and 1D). Interestingly, overexpression of GFP-CYLD-C/S decreased the level of activated RhoA, suggesting a dominant negative effect (Fig. 1C and 1D). These results indicate that CYLD stimulates RhoA activity in a deubiquitinase activity-dependent manner.

**Figure 2:**
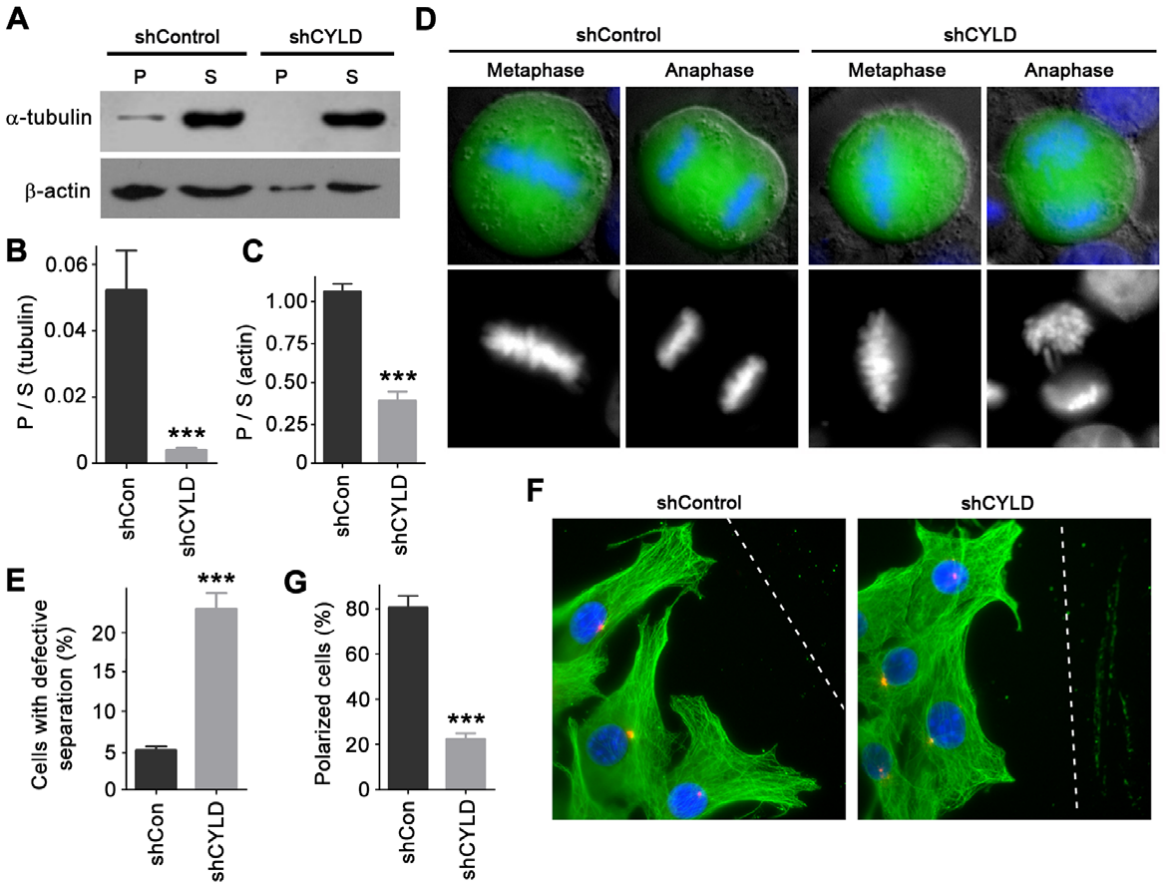
Cytoskeletal rearrangement, chromosome separation, and cell polarization are altered in CYLD-depleted cells. (A) Cells were transfected with control or CYLD shRNA, and the depolymerization and regrowth of microtubules and microfilaments were examined by measuring the amount of polymeric (P) and soluble (S) forms of tubulin and actin with immunoblotting. (B, C) Experiments were performed as in panel A, and the ratio of polymeric to soluble forms of tubulin or actin was quantified. (D) Cells were transfected with GFP together with control or CYLD shRNA, and stained with the DNA dye DAPI. Metaphase and anaphase cells were then analyzed by fluorescence microscopy. (E) Experiments were performed as in panel D, and the percentage of cells with defective chromosome separation was quantified. (F) Cells transfected with control or CYLD shRNA were scratched, and cells were fixed 2 hours later and stained with anti-α-tubulin antibody, anti-γ-tubulin antibody, and DAPI to visualize microtubules (green), centrosomes (red), and nuclei (blue), respectively. Cells with centrosomes localized between the nuclei and the leading edge were defined as polarized cells. Broken white lines indicate the wound direction. (G) Experiments were performed as in panel F, and the percentage of polarized cells at the wound margin was quantified. ***, *p*<0.001.

Since RhoA functions in cells primarily through its regulation of the cytoskeleton [1], [2], we wondered whether this effect of RhoA is altered in CYLD-depleted cells. To test this, we depolymerized cellular microtubules and microfilaments by treatment with nocodazole and cytochalasin D, respectively. Drugs were then washed out to allow the regrowth of microtubules and microfilaments. As shown in Fig. 2A–2C, the cytoskeletal rearrangement was significantly impaired in cells transfected with CYLD shRNA. We then sought to investigate whether CYLD depletion affects chromosome separation and cell polarization, which are highly dependent on RhoA-mediated cytoskeletal rearrangement [1], [2]. We found that knockdown of CYLD expression caused a significant increase in the percentage of cells with defective chromosome separation (Fig. 2D and 2E). In addition, by scratch wound assay, we found that CYLD depletion resulted in remarkable impairment in cell polarization (Fig. 2F and 2G).

**Figure 3:**
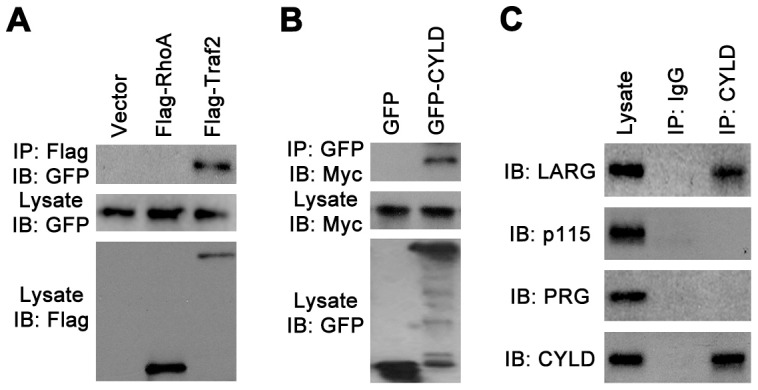
CYLD interacts with LARG but not RhoA. (A) Cells were transfected with GFP-CYLD and Flag-RhoA, Flag-Traf2, or empty vector. Immunoprecipitation and immunoblotting were then performed to analyze the interaction of CYLD with RhoA and Traf2. (B) Cells were transfected with Myc-LARG and GFP or GFP-CYLD. Immunoprecipitation and immunoblotting were then performed to analyze the interaction of CYLD with LARG. (C) Immunoprecipitation and immunoblotting were performed to analyze the interaction of endogenous CYLD with endogenous LARG, p115RhoGEF, or PDZ-RhoGEF (PRG).

We then investigated whether CYLD promoted RhoA activity via an interaction between these two proteins. By immunoprecipitation, we found that GFP-CYLD did not interact with Flag-RhoA (Fig. 3A), although it could interact with Flag-tagged tumor necrosis factor receptor-associated factor 2 (Traf2) as previously reported [20]–[22]. A recent study using mass spectrometry-based proteomic approach has identified LARG as one of the 81 proteins residing in the immunopurifications of CYLD [17]. We thus studied whether CYLD promoted RhoA activity by interacting with LARG. Immunoprecipitation assays revealed that GFP-CYLD interacted with Myc-LARG (Fig. 3B). In addition, we found that endogenous CYLD associated with endogenous LARG, but not with endogenous p115RhoGEF or PDZ-RhoGEF (Fig. 3C), demonstrating a specific interaction between CYLD and LARG.

**Figure 4:**
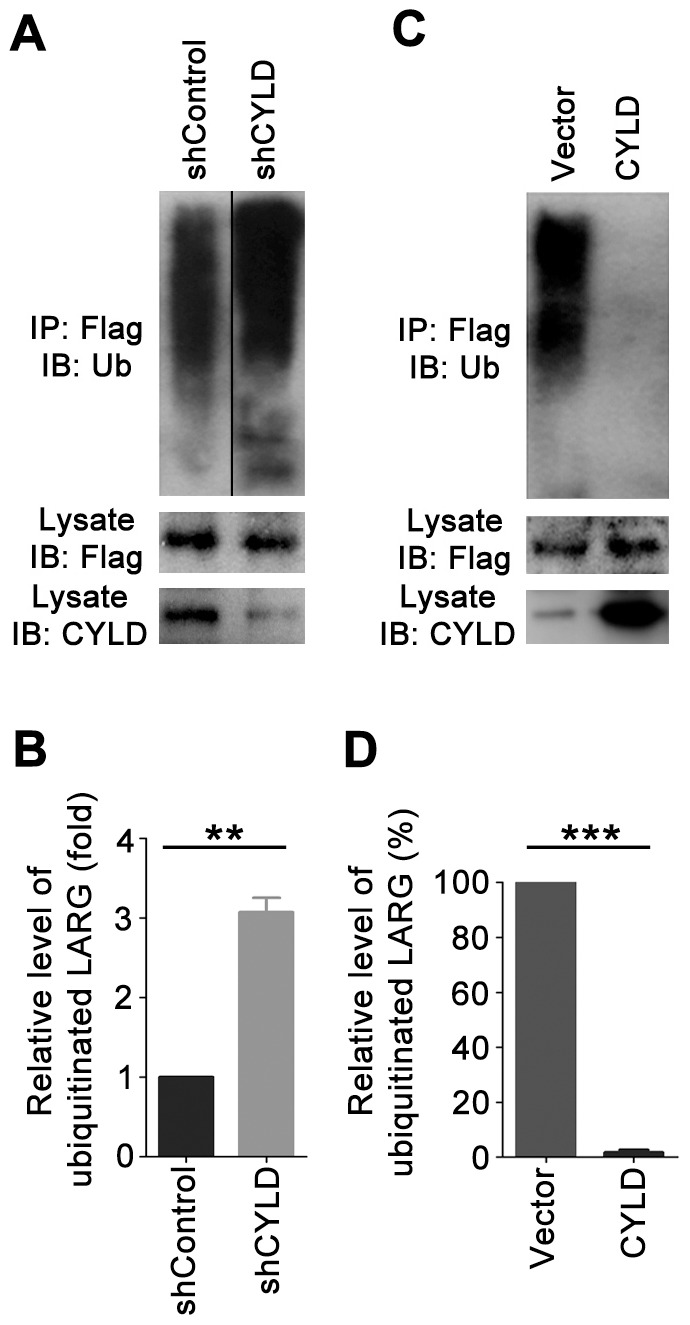
CYLD reduces the level of LARG ubiquitination. (A) 293T cells were transfected with Flag-LARG and control or CYLD shRNA. Immunoprecipitation and immunoblotting were then performed to analyze the ubiquitination of LARG. (B) Experiments were performed as in panel A, and the relative level of LARG ubiquitination was quantified. (C) Cells were transfected with Flag-LARG and CYLD or empty vector, and LARG ubiquitination was examined as in panel A. (D) Experiments were performed as in panel C, and the relative level of LARG ubiquitination was quantified. **, *p*<0.01; ***, *p*<0.001.

Since CYLD is a deubiquitinase, we wondered whether it could modulate the level of LARG ubiquitination. To investigate this, we transfected 293T cells with CYLD shRNA and Flag-LARG, and then analyzed the level of ubiquitinated Flag-LARG. We found that CYLD shRNA led to a clear increase in the ubiquitination of Flag-LARG (Fig. 4A and 4B). Conversely, overexpression of CYLD dramatically inhibited LARG ubiquitination (Fig. 4C and 4D). These data thus reveal LARG as a substrate of the CYLD deubiquitinase.

**Figure 5:**
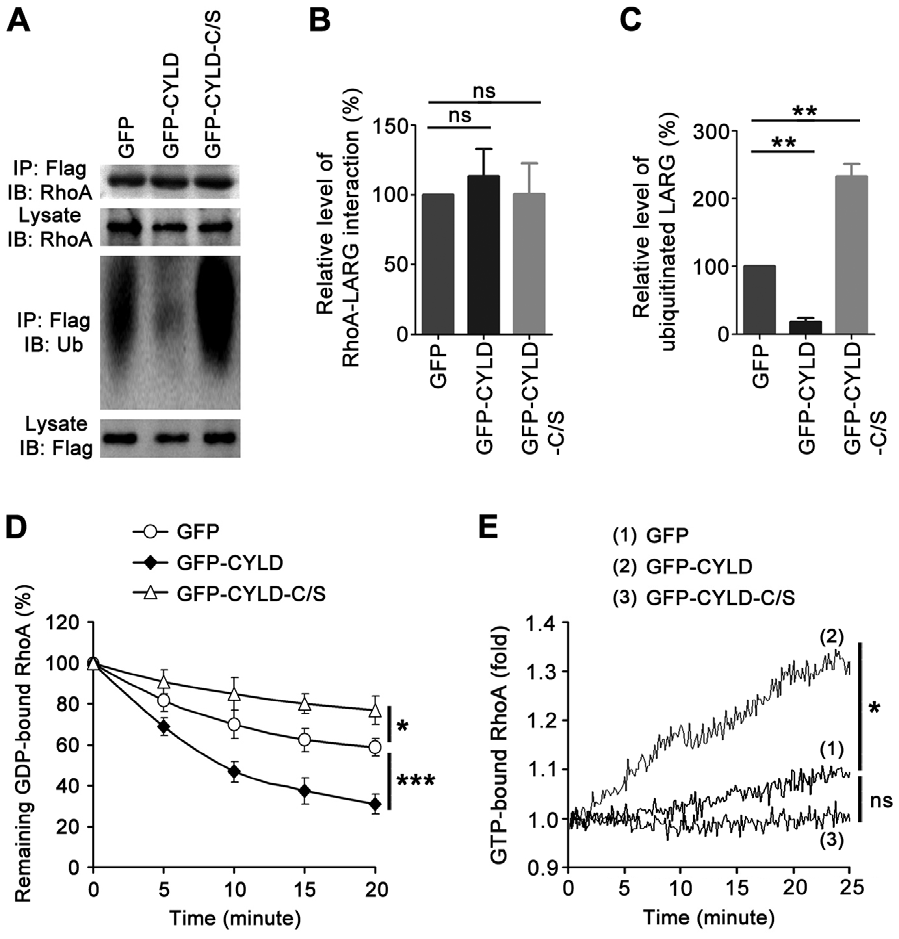
CYLD-mediated deubiquitination of LARG enhances its ability to promote GDP/GTP exchange on RhoA. (A) Cells were transfected with Flag-LARG and GFP, GFP-CYLD, or GFP-CYLD-C/S. Immunoprecipitation and immunoblotting were then performed to analyze the interaction of Flag-LARG with RhoA and the ubiquitination of LARG. (B) Experiments were performed as in panel A, and the relative level of RhoA-LARG interaction was quantified. (C) Experiments were performed as in panel A, and the relative level of LARG ubiquitination was quantified. (D) GST-RhoA preloaded with mant-GDP was incubated with Flag-LARG immunoprecipitates, and the percentage of remaining GDP-bound RhoA was determined by measuring the change in GDP fluorescence. (E) GST-RhoA preloaded with GDP was incubated with Flag-LARG immunoprecipitates in a GEF reaction buffer containing mant-GTP, and the percentage of GTP-bound RhoA was determined by measuring the change in GTP fluorescence. *, *p*<0.05; **, *p*<0.01; ***, *p*<0.001; ns, not significant (*p*≥0.05). In panels D and E, the *p* values refer to the end-time data points.

CYLD is a deubiquitinase specific for the deconjugation of lysine-63 (K63)-linked polyubiquitin chains, which often modulate protein-protein interactions [10]. We thus hypothesized that CYLD-mediated deubiquitination of LARG might enhance its interaction with RhoA. To test this possibility, we transfected 293T cells with Flag-LARG and GFP, GFP-CYLD, or GFP-CYLD-C/S, and then analyzed the interaction of Flag-LARG with RhoA. Surprisingly, overexpression of GFP-CYLD or GFP-CYLD-C/S did not obviously affect the LARG-RhoA interaction (Fig. 5A and 5B), although LARG ubiquitination was clearly decreased by GFP-CYLD and increased by GFP-CYLD-C/S (Fig. 5A and 5C).

We then sought to investigate whether CYLD-mediated deubiquitination of LARG could promote RhoA activity by enhancing the GEF activity of LARG. To assess this possibility, we transfected 293T cells with Flag-LARG and GFP, GFP-CYLD, or GFP-CYLD-C/S, and then measured GDP release from RhoA and GTP binding to RhoA using Flag-LARG immunoprecipitates and purified GST-RhoA preloaded with GDP. Fluorescent GDP or GTP was used to monitor the rate of GDP release or GTP binding. As shown in Fig. 5D and 5E, Flag-LARG effectively promoted the GDP/GTP exchange on RhoA. Importantly, the activity of LARG to mediate the GDP/GTP exchange was dramatically increased by GFP-CYLD and decreased by GFP-CYLD-C/S (Fig. 5D and 5E), suggesting that CYLD-mediated deubiquitination of LARG could enhance its GEF activity.

**Figure 6:**
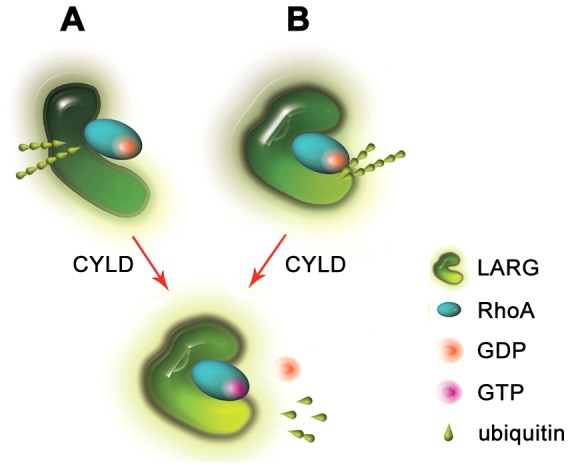
Proposed model for how CYLD-mediated deubiquitination of LARG enhances the GDP/GTP exchange on RhoA.

Taken together, our data provide the first evidence that CYLD deubiquitinates LARG and increases its ability to catalyze the GDP/GTP exchange on RhoA. At present, the precise mechanism of how CYLD-mediated deubiquitination of LARG enhances its GEF activity remains elusive. It is possible that removal of the polyubiquitin chain(s) from LARG may change its conformation so that its enzymatic active site could be exposed to RhoA (Fig. 6A). It is also possible that deubiquitination of LARG may simply remove the polyubiquitin chain barrier to facilitate the GDP/GTP exchange on RhoA (Fig. 6B). Certainly, alternative mechanisms may exist for the action of CYLD in modulating the LARG-RhoA complex.

CYLD is known to regulate a variety of cellular events primarily through its deubiquitinase activity [8]–[10]. Notable substrates of CYLD include NF-κB essential modulator (NEMO), Traf2, Traf6, Bcl3, and Rip1, which are components of the NF-κB signaling pathway. By negative regulation of NF-κB signaling, CYLD plays an important role in cell proliferation, cell cycle progression, cell survival, and many other processes [8]–[10]. CYLD has also been reported to negatively regulate innate antiviral responses through deubiquitination of retinoic acid inducible gene-I (RIG-I) [23]. In the present study, we show that CYLD deubiquitinates LARG, thus adding LARG to the growing list of CYLD substrates. Although it remains to be investigated whether CYLD causes the disassembly of K63-linked polyubiquitin chains on LARG, our findings suggest that the LARG-RhoA signaling pathway may play a role in various CYLD-mediated cellular events.
